# Is the Use of a Drain for Thyroid Surgery Realistic? A Prospective Randomized Interventional Study

**DOI:** 10.1155/2013/285768

**Published:** 2013-05-30

**Authors:** Ugur Deveci, Fatih Altintoprak, Mahmut Sertan Kapakli, Manuk Norayk Manukyan, Rahmi Cubuk, Nese Yener, Abut Kebudi

**Affiliations:** ^1^General Surgery Department, School of Medicine, Maltepe University, 34843 Istanbul, Turkey; ^2^General Surgery Department, School of Medicine, Sakarya University, 54100 Sakarya, Turkey; ^3^Radiology Department, School of Medicine, Maltepe University, 34843 Istanbul, Turkey; ^4^Pathology Department, School of Medicine, Maltepe University, 34843 Istanbul, Turkey

## Abstract

*Background*. The use of a suction drain in thyroid surgery is common practice in order to avoid hematomas or seromas. The aim of this study was to determine the efficacy of routine drainage after thyroid surgery. *Methods*. In this prospective randomized trial, 400 patients who underwent either a total thyroidectomy or lobectomy for thyroid disorders were randomly allocated to either the nondrainage (group 1) or the drainage (group 2) group. The volume of fluid collection in the operative bed, postoperative pain, complications, and length of hospital stay were then recorded. *Results*. Both groups were homogeneous according to age, gender, thyroid volume, type of procedure performed, and histopathological diagnosis. After assessment by USG, no significant difference was found between the groups in the fluid collection of the thyroid bed (*P* = 0.117), but the length of hospital stay was significantly reduced in group 1 (*P* = 0.004). *Conclusions*. In our experience, the use of drain for thyroid surgery is not a routine procedure. However, it should be used in the presence of extensive dead space, particularly when there is retrosternal or intrathoracic extension, or when the patient is on anticoagulant treatment. This trial was registered with clinical Trials.gov NCT01771523.

## 1. Introduction

It is believed that many surgeons use a drain following thyroid surgery to obliterate the dead space and evacuate collected blood and serum. This is further reinforced by the fact that postoperative drains usually yield fluid. Hemorrhage can be life threatening, thus necessitating an immediate reoperation. This fear prompts surgeons to use a routine drain after any type of thyroid surgery. Although the rate of bleeding might increase in a subtotal thyroidectomy due to vascularized remnant tissue, postoperative bleeding is actually quite rare and occurs in only 0.3–1% of patients after a thyroidectomy [1, 2]. Many studies have suggested that drains may be blocked with clotted blood; therefore, the surgeon is not alerted even if major bleeding occurs [1–4]. In addition, numerous studies have also failed to show any benefits of drainage in thyroid surgery [3–5]. Hence, the goal of this prospective study was to evaluate the necessity of drainage after thyroid surgery.

## 2. Patients and Methods

This interventional, randomized, double-blind, prospective study was approved by the local ethics committee at the Turkish Ministry of Health, and informed consent was obtained from all the patients. The subjects were 400 patients who underwent thyroid surgery between January 2010 and January 2012. These patients were randomized via a computer-generated random number table into two groups according to whether or not drains were inserted at the time of surgery. Group 1 consisted of 200 patients without drains and group 2 consisted of 200 patients with drains. The indications for surgery, procedures performed, local complications (infection, seroma, bleeding, hematoma, laryngeal nerve palsy, and hypoparathyroidism), necessity for reoperation, and length of hospital stay were recorded for all of the participants. Those with substernal goiter or malignant disease requiring lymphatic dissection were excluded from the study. In addition, nondifferentiated cancer patients and those undergoing anticoagulant therapy were also not included. 

**Figure 1:**
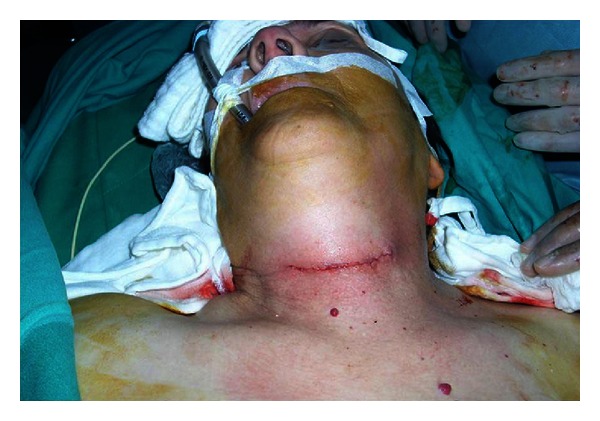
Postoperative patient in group 1.

Depending on the thyroid disorder, either a total thyroidectomy or a lobectomy plus an isthmectomy was performed. The duration of the operation was defined as the time from the first incision to the last suture placement. Wound closure was done via subcutaneous 4/0 absorbable sutures (Figure 1). Additionally a closed suction drain with negative pressure (Hemovac evacuator, Zimmer Inc., Warsaw, IN, USA) was inserted through a separate wound in the patients in group 2. An ultrasound of the neck using the B mode with a linear frequency of 7.5 MHz was performed on both groups at the postoperative 24th hour of surgery by the same radiologist. The volume of fluid collection in the operative bed was calculated by measuring the maximum diameter in three dimensions, and the fluid which collected in the drain was measured separately. The drains were removed from all of the patients after 24 hours since less than 100 cc had been collected. 

Postoperative pain was assessed according to a visual analogue scale (VAS) with scores ranging from 0 (no pain) to 10 (worst pain imaginable) at the postoperative sixth hour and postoperative first day. A standard analgesic protocol comprised of diclofenac sodium (75 mg intramuscularly) and oral paracetamol (500 mg twice a day) was then given to the patients. Differences in the pain scores were analyzed using the Mann-Whitney *U* test. Thyroid gland volumes were calculated according to the ultrasound measurements of all the patients, and the correlation between thyroid volume and volume of fluid was evaluated in the thyroid bed. The differences between the two groups were analyzed with Student's *t*-test, and a value of *P* < 0.05 was accepted as being significant. 

## 3. Results

Between January 2010 and January 2012, we performed 400 thyroidectomies in our clinic and analyzed 403 surgical interventions in these patients (mean age 46.8 ± 12.9 years; range 17–82). The male to female ratio was 1 : 7.51, and there was equal distribution in both groups based on the type of surgery and size of the nodule. The patients' characteristics are presented in Table 1, and there were no significant differences with regard to gender, age, hormonal status, or histopathological results between the two groups (*P* = 0.39, *P* = 0.45, *P* = 0.24, and *P* = 0.32, resp.). 

Similar average operating times occurred in group 1 (86.45 (50−120) ± 18.93 min) and in group 2 (88.80 (45−120) ± 21.33 min) (*P* = 0.19). However, the mean VAS score was significantly lower in group 1 than in group 2 at the postoperative sixth hour (3.64 (2−7) ± 1.06 and 4.95 (2−8) ± 1.05, resp.) (*P* = 0.002) and at the postoperative first day (2.08 (1−5) ± 0.74 and 3.09 (1−5) ± 0.77, resp.) (*P* = 0.001) (Table 2). An intramuscular analgesic was requried for all of the patients in group 2, whereas 162 (81%) of the patients in group 1 needed this medication.

The amount of fluid collection in the thyroid bed was assessed by USG for both groups at the postoperative 24th hour, and the results are shown in Table 3. Student's *t*-test was applied to detect any difference in the means of fluid collection between the groups, but there was no statistically significant difference in the volume of fluid collection (*P* = 0.117). In group 2, the amount of fluid collected in the suction drain was noted over a 24-hour period, with an average finding of 53.32 mL (range 30–90 mL/day). The mean volume of the thyroid gland was 54.31 (17.3–116.4) ± 22.48 mL and 53.72 (16.8–120.4) ± 21.61 mL in groups 1 and 2, respectively. A linear regression analysis was performed, and no significant statistical difference was seen in the amount of collection according to the volume of the thyroid gland in groups 1 and 2 (*P* = 0.73 and *P* = 0.16, resp.). The groups were also divided into toxic and nontoxic subgroups, and there was no significant difference in the volume of fluid collection (*P* = 0.192). This data is shown in Table 3. 

The complications that we observed are shown in Table 4, and the complication rates were similar between the groups (*P* = 0.43). Two cases of hematoma (1%), four cases of seroma (2%), one case of transient recurrent laryngeal nerve palsy (0.5%), one case of reaction to the silk sutures (0.5%), and eight cases of transient hypoparathyroidism (4%) occurred in group 1, whereas three cases of hematoma (1.5%), three cases of seroma (2%), two cases of wound infections (1%), one case of persistent single-side recurrent laryngeal nerve injury (0.5%), two cases of reaction to the silk sutures (1%), and six cases of transient hypoparathyroidism (3%) were present in group 2 (*P* = 0.56). Two of patients in group 1 and three in group 2 required single aspiration due to seroma. In addition, one patient in group 1 and two more in group 2 needed surgical intervention for postoperative bleeding and hematoma. The alerting symptom was the sudden increase in neck volume with dyspnea. In the second intervention, a suction drain was inserted and then removed at the postoperative 24th hour. 

The average length of hospital stay was 1.10 (1−3) ± 0.33 days for group 1 and 1.53 (1−6) ± 0.80 days for group 2, and there was a statistically significant difference noted after conducting an analysis with a two-sample *t*-test (*P* = 0.004). The mean follow-up period was 23.60 (12−36) ± 7.51 months. In the end, we concluded that the presence or absence of the drain did not contribute significantly to the postoperative complications.

## 4. Discussion 

Drains have been traditionally used in most of the surgical procedures involving the thyroid, and there is limited evidence to suggest that they provide any benefit [4–7]. Our study failed to show any advantage in the routine use of the drain after thyroid surgery. Arterial bleeding near the trachea leads to decreased space, which subsequently compresses the airway and produces significant edema in the soft tissues of the larynx and pharynx. All this causes in the syndrome known as suffocating hematoma, and immediate treatment and surgical revision in the operating theater are then required. This complication appears very infrequently, with figures ranging from 0.3 to 2.5%; however, when it does exist, it presents a big challenge for both the surgeon and the anesthesiologist [8–10]. The risk is greater in patients with intrathoracic goiter and those with Graves' disease [10]. Suffocating hematoma tends to appear between two and six hours after surgery, and most patients report coughing, vomiting, or nausea prior to the hemorrhage. Possible causes for this complication include displacement of an improperly applied suture, the opening of a vessel in which diathermia was used for coagulation, or “drooling” of an area that has been improperly cauterized [10]. 

In our series, postoperative bleeding occurred in three patients (two in group 1 and one in group 2) two hours after surgery. These patients presented with dyspnea and neck swelling, although the drain was clean. Surgical drains neither prevent this complication from occurring nor contribute to early detection [3–6, 10]. In fact, hemorrhage can appear, and the container may be empty because the blood has clotted inside the drain. Bandages do not reduce the risk of hemorrhage either. They keep blood from collecting in the subcutaneous plane, but the blood may dissect the deep plane to the prethyroid musculature in the paratracheal region, leading to compression of the airway at that level [10]. 

Two large nonrandomized studies of 250 and 400 patients have also documented that the use of drains after thyroid surgery produces no benefits [8, 11]. In our study, there was an absence of fluid in the thyroid bed on USG, but it was present in the suction drain. This could be caused by the drain itself, since by virtue of the inflammation caused by their presence, they may actually increase the drainage. In addition, the vacuum created by the negative suction of the drain may prevent the lymphatics from sealing off, thus causing an increase in seroma formation and drainage [4, 12]. Furthermore, a possible relationship between drain insertion and infective complications has been observed in some studies [1, 11, 13]. However, we found no relationship between wound infection and drain usage in our study (Table 4). Two studies also investigated the relationship between drain insertion and postoperative pain [1, 14], and the authors noted an approximate 50% reduction in the VAS score in the group in which no drains were used. We obtained similar results in our study. These results indicate that drain insertion might be directly associated with higher levels of postoperative discomfort due to increased pain. This would be reflected by patient satisfaction and early discharge independent of any complications. Morrissey et al. demonstrated that thyroid surgery without the use of a drain decreases the length of hospital stay while producing no increase in patient morbidity [15]. We also observed that performing a thyroidectomy without the use of drain decreased the length of hospital stay.

Meticulous hemostasis and an adequate surgical technique are the keys for avoiding hemorrhage and hematoma formation. Herranz and Latorre suggested using a drain in cases in which there is extensive dead space or retrosternal goiters and also noted that routine drainage of the thyroidectomy bed is not effective in decreasing the rate of postoperative complications after thyroid surgery [10]. In a meta-analysis, Corsten et al. concluded that the use of suction drains in thyroid surgery to prevent postoperative hematoma is not evidence based [16]. Furthermore, Khanna et al. reported no significant differences between patients with drains and those without in the collection of the thyroid bed when this was assessed by postoperative USG [5]. In this study, we also demonstrated that the use of a drain for thyroid surgery was not effective for decreasing the rate of complications associated with toxic thyroid disorders. Additionally, a total thyroidectomy performed without a drain is safe and effective for patients with Graves' disease or toxic goiter. 

Most studies have revealed that drainage is unnecessary after routine thyroid surgery [1, 3–5, 13, 14]. However, many of these studies had a small number of patients and a retrospective design. This prospective study contains the largest number of patients on this topic in the literature. We do not routinely use drains in our department for thyroid surgery. We only recommend that they should be used in thyroidectomies for patients with substernal goiter and nondifferentiated cancer along with those who are undergoing anticoagulant therapy or require lymphatic dissection. We believe that thyroidectomies without drains are safe for differentiated thyroid cancer, Graves' disease, and other toxic goiters. 

## 5. Conclusion

Our randomized prospective study verified that routine drain placement after thyroid surgery is not necessary nor is it effective in decreasing the rate of postoperative complications. Meticulous hemostasis and attention to finer details during surgery are more important for achieving this goal. Thyroid surgery without the drain decreases the length of hospital stay without increasing patient morbidity. Hence, we found no positive evidence that the use of drains improves patient outcomes. 

## Figures and Tables

**Table 1:** Patient characteristics.

	Group 1	Group 2
Age	46.80 (17–82) ± 12.90	44.33 (20–79) ± 12.01
Gender (male/female)	21/179	26/174
Type of surgery (total thyroidectomy/lobectomy)	164/36	172/28
Diagnosis		
Benign	178 (89%)	184 (92%)
Malign	22 (11%)	16 (8%)
Toxic	24 (12%)	28 (14%)
Non-toxic	176 (88%)	172 (86%)

**Table 2:** Operative and postoperative values of the patients.

	Group 1	Group 2	*P *
*Operating time (min)	86.45 (50–120) ± 18.93	88.80 (45–120) ± 21.33	0.19
*Thyroid volume (mL)	54.31 (17.3–116.4) ± 22.48	53.72 (16.8–120.4) ± 21.61	0.80
**Postoperative sixth hour VAS	3.64 (2–7) ± 1.06	4.95 (2–8) ± 1.05	0.002
**Postoperative first day VAS	2.08 (1–5) ± 0.74	3.09 (1–5) ± 0.77	0.001
*Hospital stay (day)	1.10 (1–3) ± 0.33	1.53 (1–6) ± 0.80	0.04

VAS: visual analog scale. The data is presented as mean (min–max) ± SD. *Student's *t*-test was used for assessment. **The Mann-Whitney *U* test was used for assessment.

**Table 3:** Volume of fluid collection in the groups as assessed by USG.

Group 1 (*N* = 200)		Group 2 (*N* = 200)	
4.09 (0–25) ± 6.08 mL		3.64 (0–30) ± 5.07 mL	
*P* = 0.11			

Group 1		Group 2	
Toxic (*N* = 24)	Nontoxic (*N* = 176)	Toxic (*N* = 28)	Nontoxic (*N* = 172)

4.37 (0–18.4) ± 4.24	4.39 (0–25) ± 5.97	3.81 (0–18.3) ± 5.41	3.83 (0–30) ± 5.05
*P* = 0.19		*P* = 0.16	

The data is presented as mean (min–max) ± SD. Student's *t*-test was used for assessment.

**Table 4:** Postoperative complications (*P* > 0.05*).

	Group 1	Group 2
Hematoma	2 (1%)	3 (1.5%)
Seroma	4 (2%)	3 (1.5%)
Wound infection	0 (0%)	1 (0.5%)
Suture reaction	1 (0.5%)	2 (1%)
Transient recurrent nerve praxy	1 (0.5%)	0 (0%)
Persistant recurrent nerve injury	0 (0%)	1 (0.5%)
Transient hypoparathyroidism	8 (4%)	6 (3%)
Persistant hypoparathyroidism	0 (0%)	0 (0%)

The data is presented as the number of patients with percentiles in parenthesis. A chi-square test was used for all of the complications. *P value is presented for the total number of complications.
